# Oxazinethione Derivatives as a Precursor to Pyrazolone and Pyrimidine Derivatives: Synthesis, Biological Activities, Molecular Modeling, ADME, and Molecular Dynamics Studies

**DOI:** 10.3390/molecules26185482

**Published:** 2021-09-09

**Authors:** Magda H. Abdellattif, Mohd Shahbaaz, M. M. H. Arief, Mostafa A. Hussien

**Affiliations:** 1 Department of Chemistry, College of Science, Taif University, P.O. Box 11099, Taif 21944, Saudi Arabia; 2 South African Medical Research Council Bioinformatics Institute, University of Western Cape, Private Bag X17, Bellville, Cape Town 7535, South Africa; mohammed@sanbi.ac.za; 3 Laboratory of Computational Modelling of Drugs, South Ural State University, 76 Lenin Prospects, 454080 Chelyabinsk, Russia; 4 Chemistry Department, Faculty of Science, Benha University, Benha 13511, Egypt; mohamed.arief@fsc.bu.edu.eg; 5 Department of Chemistry, Faculty of Science, Port Said University, Port Said 42521, Egypt; maabdulaal@kau.edu.sa; 6 Department of Chemistry, Faculty of Science, King Abdulaziz University, P.O. Box 80203, Jeddah 21589, Saudi Arabia

**Keywords:** oxazines, pyrimidine, pyrazole, one-pot synthesis, molecular docking, ADME

## Abstract

In this study, we used oxazinethione as a perfect precursor to synthesize new pyrimidine and pyrazole derivatives with potent biological activities. Biological activities were determined for all compounds against *A. flavus, E. coli, S. aureus, and F. moniliform*. Compounds **3**, **4a-b**, and **5** exhibited higher activities toward *A. flavus, E. coli, S. aureus, and F. moniliform*; this was indicated through the MIC (minimum inhibitory concentration). At the same time, anticancer activities were determined through four cell lines, Ovcar-3, Hela, MCF-7, and LCC-MMk. The results obtained indicated that compound 5 was the most potent compound for both cell lines. Molecular docking was studied by the MOE (molecular operating environment). The in silico ADME of compounds **2** and **5** showed good pharmacokinetic properties. The present research strengthens the applicability of these compounds as encouraging anticancer and antibacterial drugs. Moreover, JAGUAR module MD simulations were carried out at about 100 ns. In addition, spectroscopic studies were carried out to establish the reactions of the synthesized structure derivatives.

## 1. Introduction

**Figure 1:**
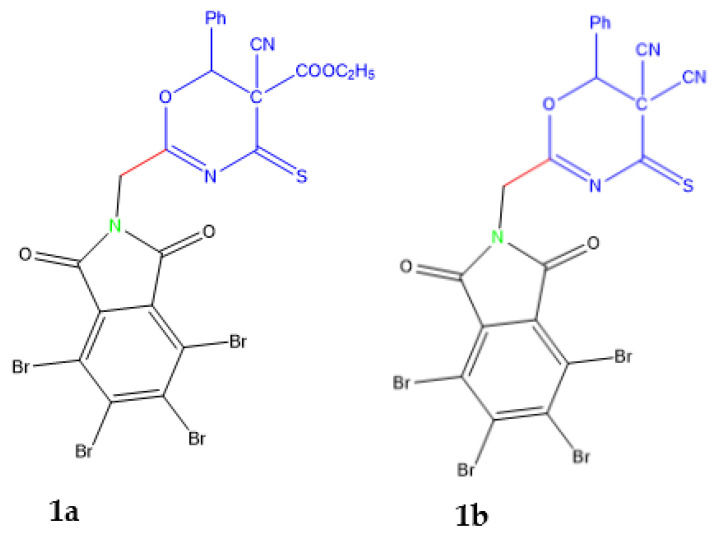
Compounds **1a** and **1b** are indexed by Magda et al. [1].

Oxazinethione is a bright nucleus in many pharmacological studies and applications. One of oxazinethione is compound **1a**-**b** which was prepared in our laboratory Figure 1. Therefore, oxazine derivatives were studied as an antimicrobial with promising results [1]; these nuclei. In addition, some derivatives of oxazinethione have been used as an anticancer, anti-inflammatory, analgesic, and many pharmacological activities [2,3].

Moreover, oxazinethione was reported to have antimicrobial activities [4]. The new value in this research is the use of oxazine as a precursor for pyrimidine derivatives, and these new derivatives have essential biological activities [5]. An easy way of synthesizing and studying the biological activities of oxazine pyrimidine and oxazine pyrazole derivatives was reported with promising results [6]. Many drugs containing the oxazinethione moiety serve a different medical purpose, such as Timolol as an antihypertensive drug and reboxetine, which treats significant depression. More than 200 pyrimidine derivatives have been used as antimicrobial agents with other mono-, di-, tri-, and tetrasubstituted classes [7]. In addition, in vitro studies of the antimicrobial activities of pyrimidine derivatives can facilitate the development of more potent and effective antimicrobial agents [8]. Some pyrimidines, especially minoxidil, are vasodilating antihypertensive agents used for resistant hypertension that is symptomatic or has caused end-organ damage, which also acted on prostaglandin G/H synthase [9]. Our new research describes oxazinethione as a precursor, developing pyrimidine and pyrazole derivatives as antimicrobial and anticancer drugs, and they were tested against four cell lines, Ovcar-3 (ovarian), Hela (cervical Hela), MCF-7 (breast), and LCC-MMk (normal cell). Molecular docking was used to predict and prove the biological studies obtained by MOE, validated by general proteins using JAGUAR modules. ADMET studies and molecular dynamic studies indicated the prediction and validation of the activity of the synthesized compounds. The use of computational studies is one of the modern techniques that help describe the pharmacological properties of the synthesized compounds. The consequence of the present research strengthens the applicability of these compounds as encouraging anticancer and antibacterial drugs that could help medicinal chemists and pharmaceuticals further design and synthesize more effective drug candidates [10,11,12,13,14,15,16].

## 2. Results and Discussion 

### 2.1. Chemistry

**Scheme 1:**
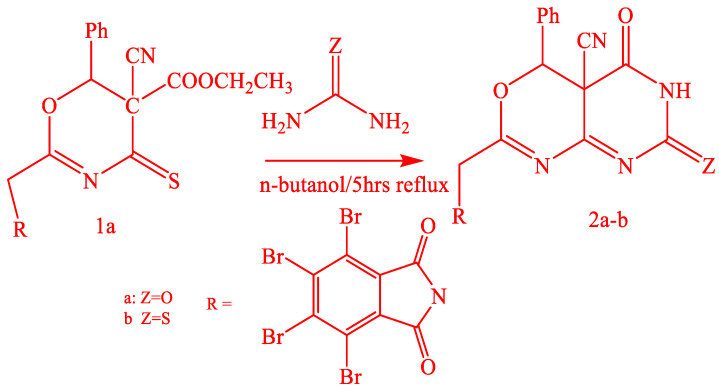
Synthesis of compounds **2a-b**.

**Scheme 2:**
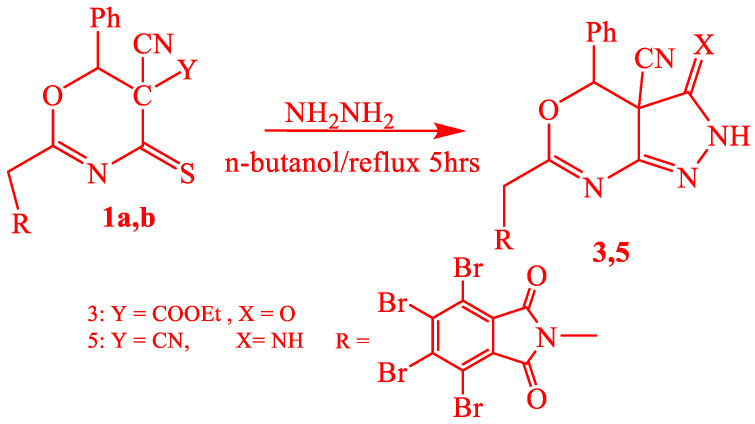
Synthesis of compounds **3** and **5**.

Sensitized oxazinethione [1] is the precursor of the new pyrimidine derivatives: (Scheme 1 and Scheme 2), pyrazolone derivatives, oxazinethione.

Oxazinethione derivative (**1a**) reacted with urea and thiourea in n-butanol by boiling, which gave 2-(tetrabromophthalimidomethyl-6-phenyl-5-cyano-1,3-oxazino[4,5-e]-1,3-pyrimidine-3-[H]-2,4-dione (**2a**) and 2-(tetrabromophthalimidomethyl-6-phenyl-5-cyano-1,3-oxazino[4,5-e]-1,3-pyrimidine-3-[H]-2-thione-4-one (**2b**). The structures of both (**2a** and **2b**) were proved by analytical data, and the IR spectra of (**2a**) and (**2b**) exhibited absorptions at 1720–1710 cm^−1^ (CO), 2230–2220 cm^−1^ (CN), 1200–1190 cm^−1^ (CS), and 3380–3370 cm^−1^ (NH). The ^1^HNMR of **2b** showed signals at δ ppm 3.7 (2H, CH_2_), 7.2–7.8 (6H, AH+NH), and (1H, phCH) *J* = 5.3. Compound (**1a**) also reacted with hydrazine hydrate in boiling n-butanol, resulting in 2-(tetrabromophalimidmethyl)-6-phenyl-5-yano-1,3-oxazino[4,5-d]-1,2-pyrazole2[H]-3-one (**3**), Scheme 2.

**Scheme 3:**
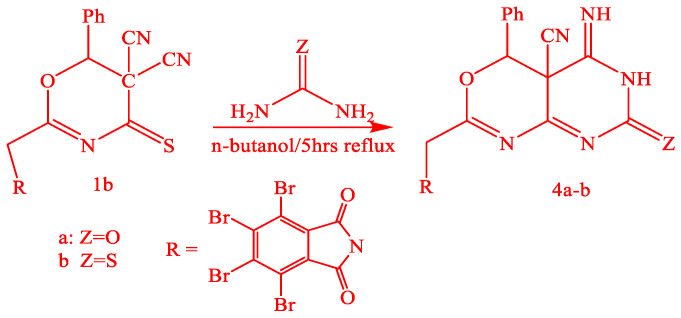
Synthesis of compounds **4a-b**.

The compound (**3**) structure was proved by analytical data, and the IR spectra exhibited absorptions at 1715 cm^−1^ as CO, 2205 cm^−1^ as the presence of CN, and 3154–3350 cm^−1^ as the presence of NH. Oxazinethione derivative (**1b**) reacted with urea and thiourea in n-butanol with boiling 2-(tetrabromophthalimidomethyl-6-phenyl-5-cyano-1,3-oxazino[4,5-e]-1,3-pyrimidine-3-[H]-2-one-5-imide (**4a**) and 2-(tetrabromophthalimidomethyl-6-phenyl-5-cyano-1,3-oxazino[4,5-e]-1,3-pyrimidine-3-[*H*]-2-thione-4-imide (**4b**), Scheme 3.

**Figure 2:**
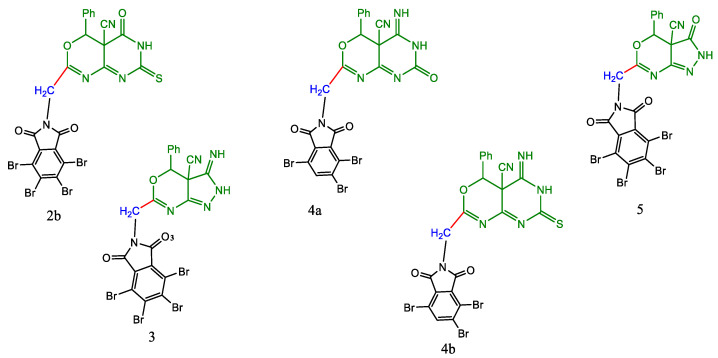
The synthesized compounds **2a**, **3**, **4a**, **4b**, and **5**.

Analytical data proved the structure of compounds (**4a-b**), and the IR spectra of **4a** and **4b** exhibited absorptions at 1278 cm^−1^ (CS), 2260 cm^−1^ (CN), 3378 cm^−1^ (NH), and 1780 and 1720 cm^−1^ (CO). The mass spectrum of (**4b**) represented a sharp characteristic molecular ion peak at *m*/*z* 758. Compound (**1b**) also reacted with hydrazine hydrate in boiling n-butanol, resulting in 2-(tetrabromophalimidmethyl)-6-phenyl-5-yano-1,3-oxazino[4,5-d]-1,2-pyrazole2[*H*]-3-imide (**5**), Scheme 2. Analytical data proved the structure of compound (**5**); it showed the IR spectrum at 3350–3310 cm^−1^ (N.H.) and 2360 cm^−1^ (CN), in addition to the coupling of carbonyl bands of cyclic imide at 1780 and 1730 cm^−1^. ^1^HNMR showed characteristic signals at (δ ppm), 3.2 (H, CH_2_), 7.2–7.8 (7H, ArH, and 2NH), and (1H, PCH). All the mechanisms of the reactions are included in Tables. While Figure 2 represents the structures of the synthesized compounds.

### 2.2. Biology

#### 2.2.1. Antimicrobial Studies

All the prepared compounds were subjected to investigations regarding Gram-positive and Gram-negative bacteria [17,18,19,20]. From the obtained biological data in Table 1, an evident elevated level of activity was demonstrated for derivatives **2a, 2b, 3, 4a, 4b**, and **5** in evaluating *A. flavus*, with similar behavior being detected with **3, 4a**, and **5**; **2a, 3, 4a, 4b,** and **5** exhibited activity toward *E. coli*, while **2a, 2b, 3, 4b**, and **5** exhibited activity toward *S. aureus*. Moreover, all of them were active in the assessment with *F. moniliform*. The results are summarized in Table 1.

#### 2.2.2. Anticancer Studies

**Figure 3:**
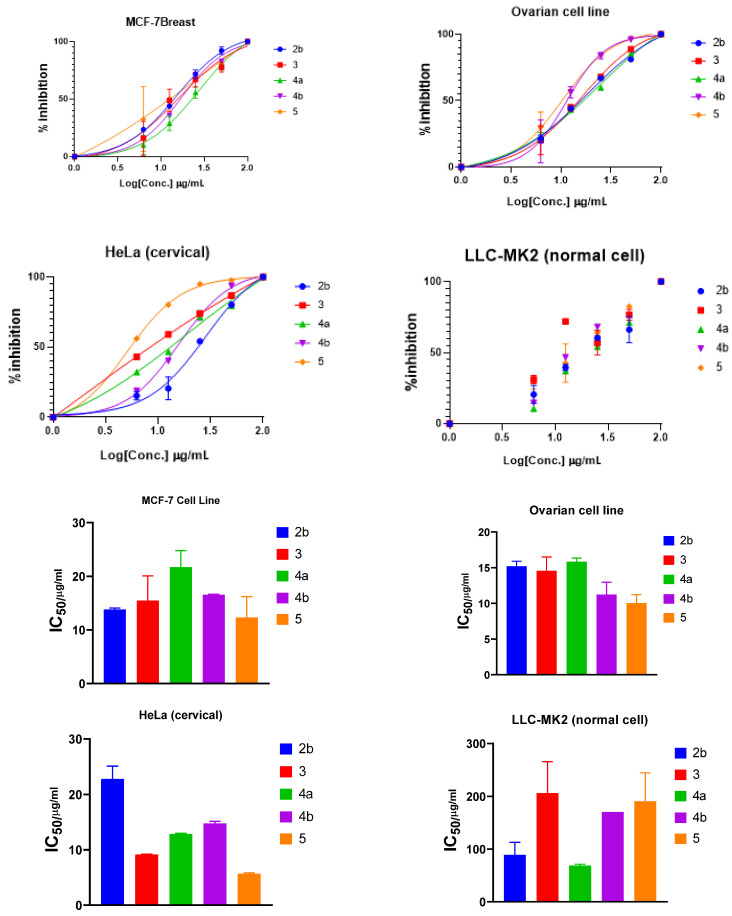
Inhibition and IC_50_ of selected compounds in DMSO solutions toward MCF-7, Hela, Ovcar-3, and LLC-MK2 cell lines.

All the synthesized compounds were tested against three cell lines, MCF-7 for breast cancer, Ovcar-3 for ovarian cancer, and Hela for cervical cancer. In addition, all the results obtained were compared and validated to the LCC-MK2 normal cell line. The results obtained in Table 2 indicated that compounds **4b** and **5** are the most potent compounds. The results obtained are illustrated in Table 2, and graphical statistical analysis is represented in Figure 3. Compound 5 showed to be the most potent and effective for the three cell lines, which posed IC_50_ values of 12.31, 5.69, and 10.05 µg/mL for MCF-7, HeLa, and ovcar-3, respectively. On the other hand, the IC_50_ of LCC-MK2 showed that these compounds are of moderate safety as most of the IC_50_ values are less than 200 µg/mL.

### 2.3. Computational Studies

#### 2.3.1. Molecular Modeling Studies Using MOE

**Figure 4:**
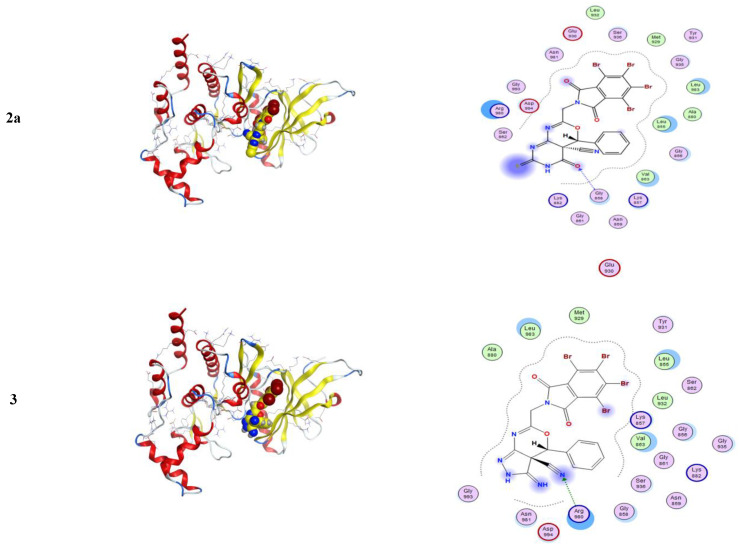

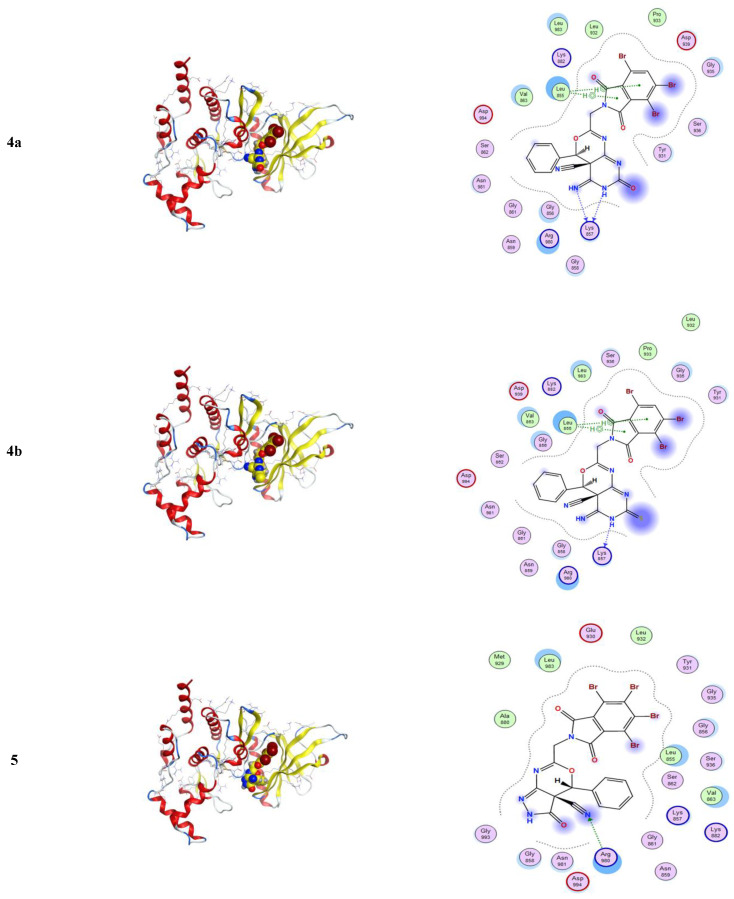
3D and 2D snapshots show the hydrophilicity interaction to the MCF-7 breast target (*3 KRR*) receptor.

Molecular docking is a computational software routinely used for understanding the protein–receptor interaction with complexes [10,21]. The docking process was carried out by simulating the interaction of the prepared compounds with three types of protein-receptors: MCF-7 breast (PDB = *3 KRR*), Ovcar-3 Ovarian cancer (PDB = *3 W2S*), and HeLa caspase (PDB = *3 V266*), which have been selected according to the literature and previous studies [22,23,24]. The docking score energies of MCF-7 (PDB = *3 KRR*), HeLa caspase (PDB = *3 V266*), and Ovarian cancer (PDB = *3 W2S*) are represented in Table 3, Table 4 and Table 5, respectively, and indicates that compound 5 is the most potent. All 3D and 2D interactions for the three targets are illustrated in Figure 2, Figure 3 and Figure 4, respectively. The docking interactions of MCF-7 (PDB = *3 KRR*), HeLa caspase (PDB = *3 V266*), and Ovarian cancer (PDB = *3 W2S*) are illustrated in Table 6, Table 7 and Table 8, respectively.

From Table 3, the docking score energies of the synthesized compounds **2a**, **3**, **4a**, **4b**, and **5** with *3 KRR* were −8.43, −8.05, −8.06, −8.1, and −8.45, respectively. From Table 4, the docking score energies of the synthesized compounds **2a**, **3**, **4a**, **4b**, and **5** with *V266* were −6.22, −7.45, −6.74, −7.15, and −7.41, respectively. Finally, from Table 5, the docking score energies of the synthesized compounds **2a**, **3**, **4a**, **4b**, and **5** with *2 W2S* were −7.7, −7.39, −7.66, −8.38, and −8.77, respectively. These results indicate that the most potent compound for the three proteins was compound **5**, while compound **5** was most potent to *2 W2S*.

**Figure 5:**
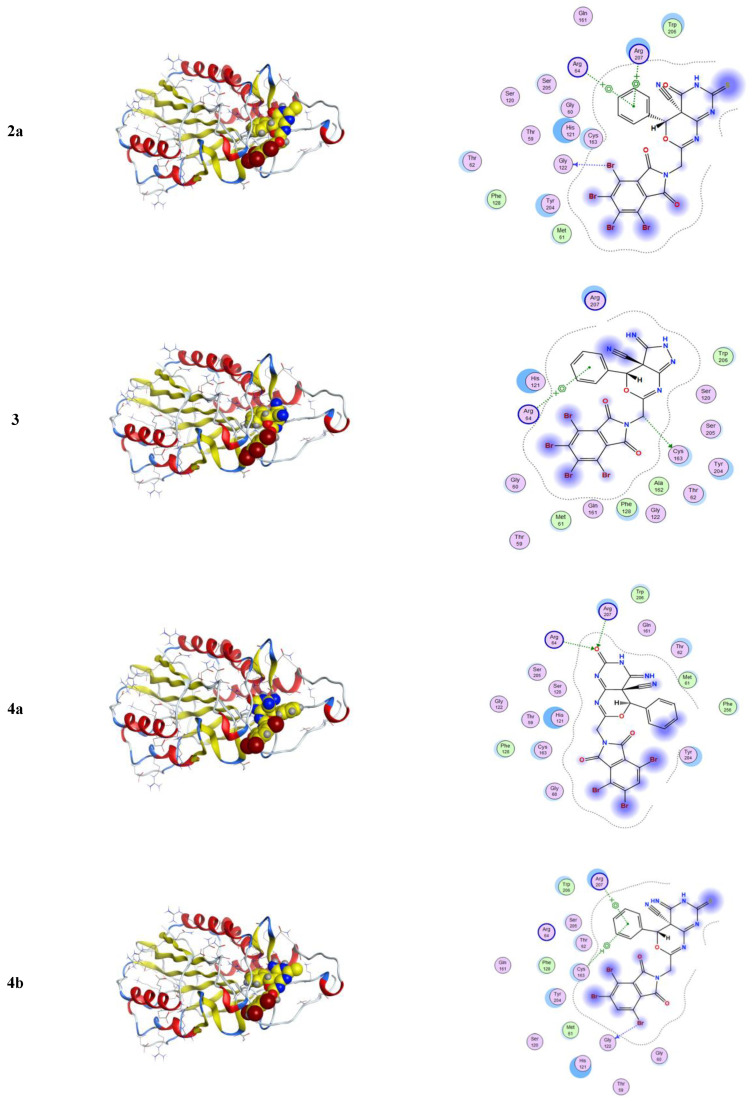

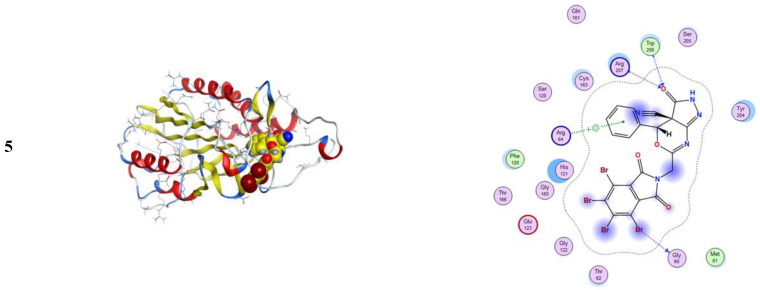
3D and 2D snapshots showing the hydrophilicity interaction to HeLa caspase3 target receptor “*V266*”.

**Figure 6:**
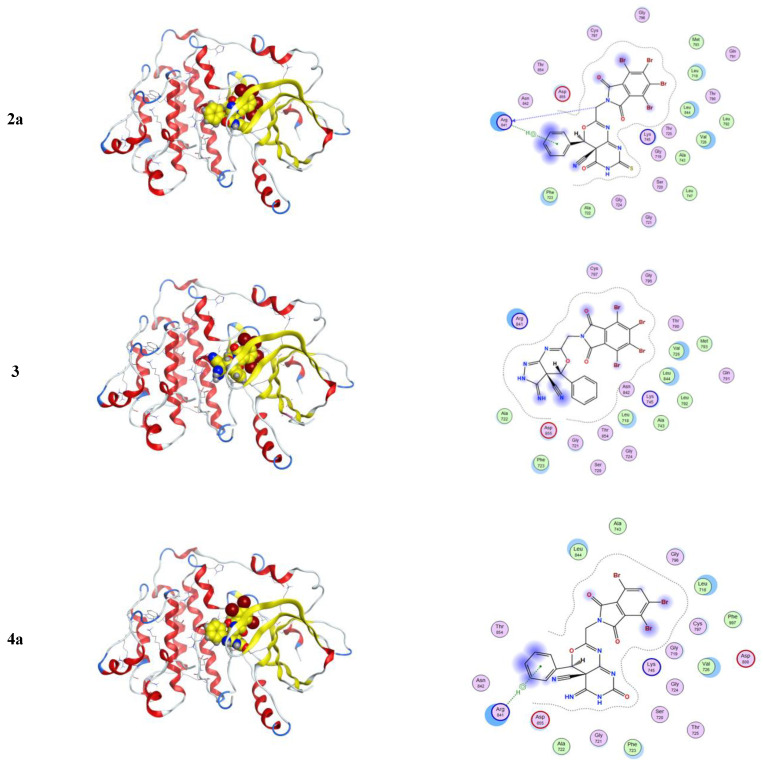

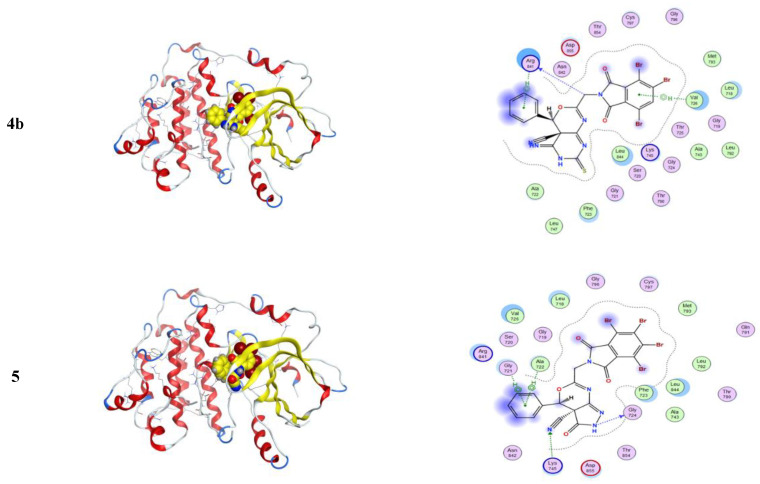
3D and 2D snapshots show the hydrophilicity interaction to the ovarian cancer target (*3 W2S*) receptor.

From Figure 4, one can notice in the case of the MCF-7 target receptor that the docking pose of the *3 KRR* protein showed that the main contribution of the activity belonged to the interaction of our compounds with the two amino acids Arg 880 and Gly 858 by a nitrogen atom of the cyanide group and carbonyl group in compounds **2a** and **5**. Meanwhile, from Figure 5, in the HeLa caspase3 target receptor and the docking pose of *V266* protein, one can also notice that the main contribution to the activity that belonged to the interaction of our compounds with the amino acids Arg 64, Arg 207, Trp206, and Gly 60 was by one of a benzene ring, carbonyl group, and bromine atom, respectively, which were represented in compound **5**. Finally, from Figure 6, in the ovarian *3 W2S* protein, the main contribution of the activity was the interaction with our compounds with the three amino acids Gly 721, Ala 722, and Lys 745 by one of a benzene ring and cyano group. These results of docking interactions for the different proteins were represented in compound **5**, as shown in Table 6, Table 7 and Table 8, respectively.

#### 2.3.2. ADME and Pharmacophore Studies

ADME (absorption, distribution, metabolism, and excretion)**,** including drug-likeness analysis, are essential in drug discovery, which accommodates the reasonable decision-making on whether or not inhibitors can be used in a biological system [21,25]. A potent antagonistic interaction of inhibitors with a receptor protein or enzyme cannot guarantee the ability of an inhibitor as a drug; therefore, ADME assessment is essential in drug development. Inhibitors with lower ADME properties and high toxicity effects on the biological systems are often the dominant explanation of most failed medicines in the clinical phase of experiments.

**Figure 7:**
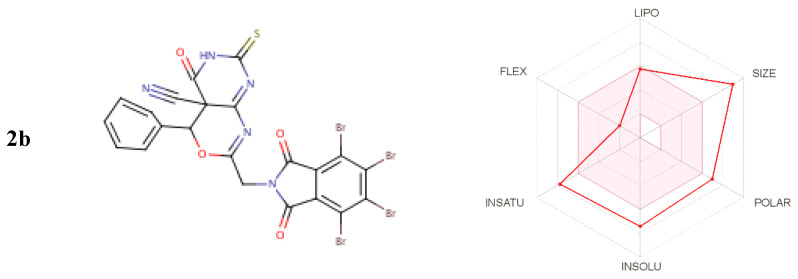

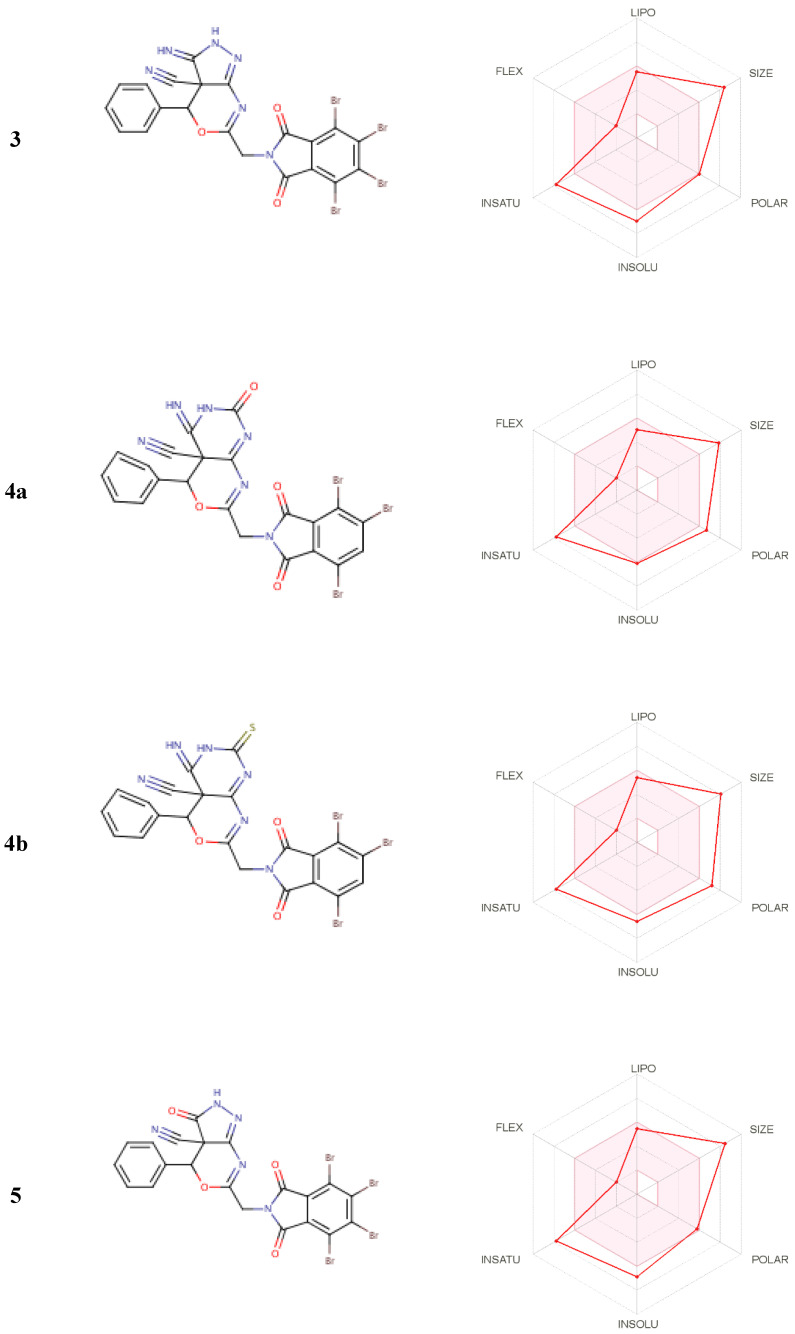
Inhibitors for biological systems.

From Figure 7, from the output of some ADME and drug-likeness properties shown in Table 9, Table 10 and Table 11, it was observed that compounds **2b** to **5** molecules have one or two violations of Lipinski’s rule, and the first violated is the molecular weight rule with 663.07–759.02 g/mol. The drug-likeness parameters are related to the aqueous solubility and intestinal permeability, determining the first step of oral bioavailability [25]. The results also showed good pharmacokinetic properties in which compounds **2b** and **5** have high gastrointestinal absorption.

#### 2.3.3. Molecular Dynamics Studies and Validations of the Methods Used

##### Analyses of the Interaction Patterns and Conformational Dynamics

**Figure 8:**
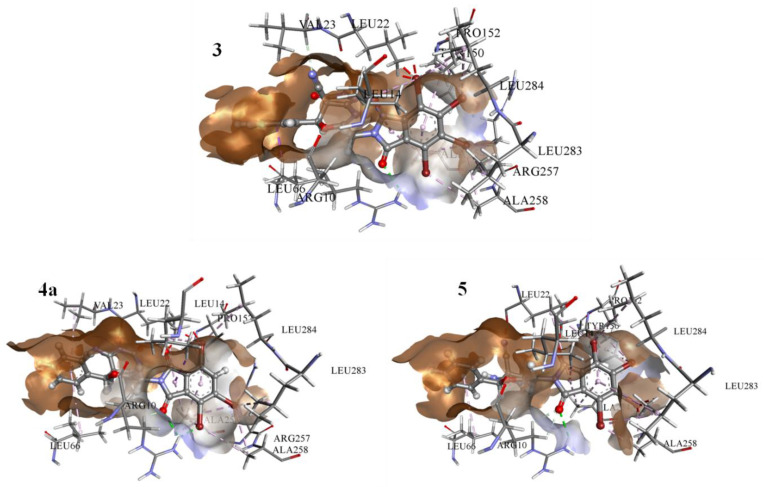
The result from docked complexes of HSA and studied inhibitors **3**, **4a**, and **5**.

The protein selected for this study is listed in Table 12. The information regarding the active site of the proteins was collected from the literature [26,27,28,29,30,31,32]. The active site of the caspase-3 was observed at Met61, His121, Phe128, Cys163, Thr166, and Tyr204, while for cyclin-dependent kinase 2 (CDK2), it involved the residues Glu81, Phe82, Leu83, Asp86, and Lys89. Similarly, for the epidermal growth factor receptor (EGFR), the crystal structure showed that the binding site was present at Val726, Ala743, Lys745, Met766, Leu777, Leu788, Thr790, Met793, Asp800, Leu844, Thr854, and Asp855. The Human B-Raf Kinase includes Ala481, Lys483, Leu514, Ile527, Trp531, Cys532, Asp594, and Phe595 in the interaction cavity, while the Human Estrogen Receptor Ligand-Binding Domain has Glu353, Leu391, Arg394, Leu402, Ile424, His524, Leu525, and Leu540. There is varied information available regarding the human serum albumin (HSA) active site in the literature and databases. Therefore, all the residues were considered, while for Human topoisomerase I, the activity was observed at Arg488, Lys532, Arg590, and His632. The highest binding affinities were observed for the HSA against the **4a**, **3**, and **5** with the free energies of binding of −9.9 kcal/mol, −9.8 kcal/mol, and −9.3 kcal/mol, respectively. All three inhibitors showed similar residue patterns with the HSA, Figure 8.

**Figure 9:**
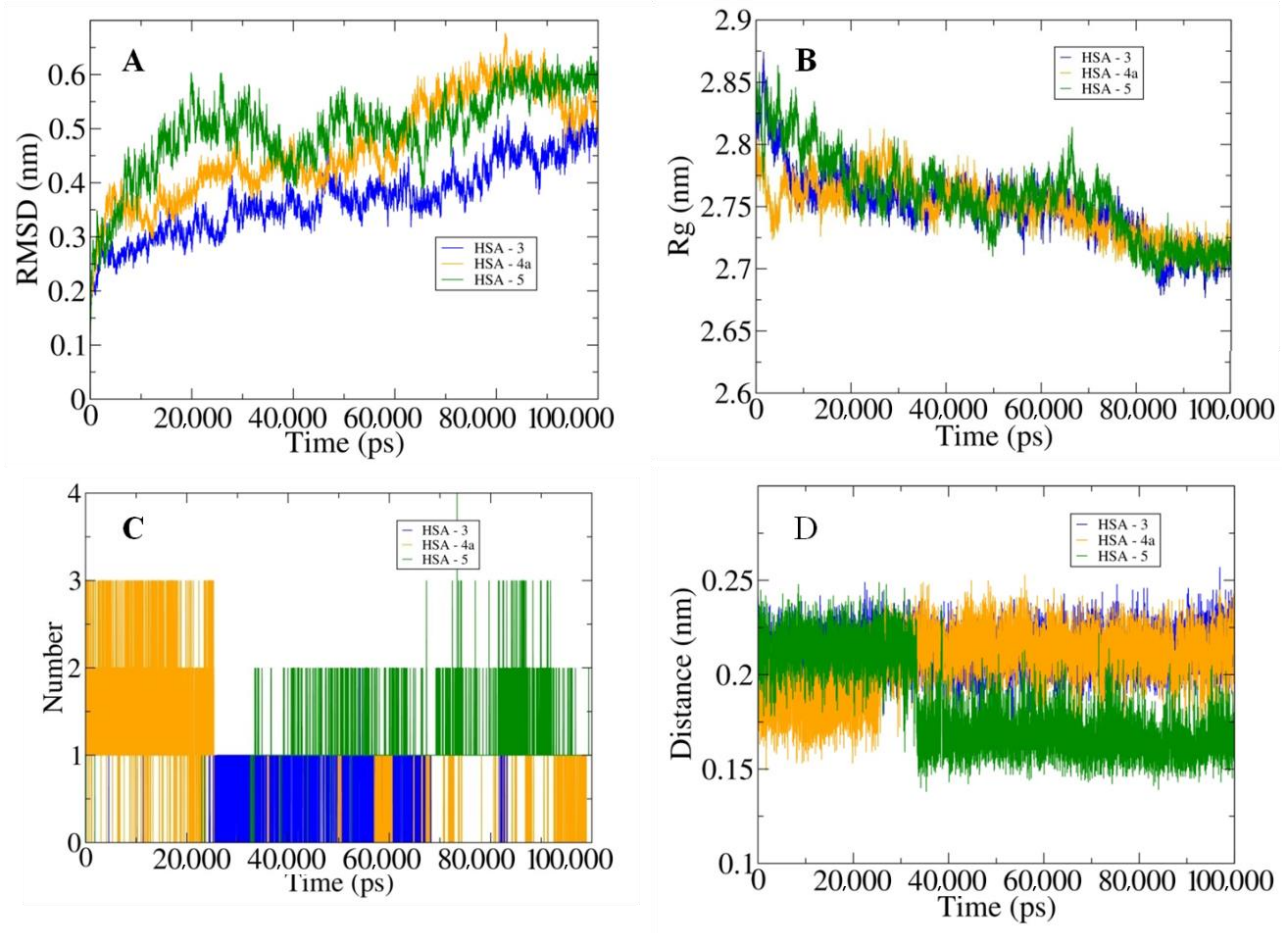
The outputs generated from 100 ns MD simulations with (**A**) illustration of the changes in the RMSD values for the three studied systems, and (**B**) changes in the Rg values. (**C**) Graph depicting the variation in the number of hydrogen bonds between HSA and docked inhibitors. (**D**) Curves showing the fluctuations in the calculated distances.

The HSA complexes with the highest binding affinities were selected for 100 ns molecular dynamics (MD) simulations. The root-mean-square deviation (RMSD) values provided the stability profile of the studied systems. One can observe that the **3a** system achieves the highest stability with the values observed between 0.3 nm and 0.5 nm compared to the other systems (Figure 9A). Estimating the degree of compactness of the studied systems in a radius of gyration (Rg) showed a similar degree of compactness for all the systems with values observed between 2.7 nm and 2.85 nm (Figure 9B).

Furthermore, we inferred the bonding patterns from the hydrogen bonding and the calculated distances between the HSA and the studied systems. Inhibitor **5** showed the highest number of H-bonds of four with HSA, and inhibitors **4** and **3** showed three and two bonds, respectively (Figure 9C). One can also observe similar behavior for the calculated distances with the least distance of around 0.15 nm between the HSA and inhibitor **5** (Figure 9D). Moreover, the MM/PBSA-based protocols were used to calculate the diverse interaction energies between the HSA and the studied inhibitors. Inhibitor **3** showed the highest binding energy of −375.922 kJ/mol, followed by inhibitors **5** and **4a** (Table 13). These observations validated the experimental findings and showed that the studied inhibitors favorably bonded to the HSA proteins and may result in the limitation of the cancer proliferation by inhibiting the activities of the proteins.

## 3. Methodology

### 3.1. Chemistry

All reactions were carried out with the exclusion of moisture. All solvents were dried. All melting points were uncorrected. The IR spectra were recorded as potassium bromide pellets on an Aldrich FT-IR spectrometer (Central lab at Faculty of Science, Benha, Ain Shams, and Cairo universities). Mass spectra were recorded using GCMS (gas chromatography-mass spectrometry) on the Shimadzu Q.P.-2010 Plus (Microanalytical center, Ain shams University), using UV light. Spectrometer spins on a Bruker DPX 400 MHz Spectro spins were used to record the ^1^HNMR (500 MHz, DMSO-*d*_6_) and ^13^CNMR (125 MHz, Chloroform-*d)* spectra. Chemical shift (d) values were stated in parts per million (ppm) using internal standard tetramethylsilane. The D_2_O exchange confirmed the exchangeable protons (OH and NH). LC-MS/MS (PerkinElmer) was used to record the mass spectra, presented as *m*/*z*. Elemental analyses were achieved at Ain Shams University on an elementary analysis system by using a PerkinElmer 240 analyzer. The purity of synthesized compounds, as well as the progress of the reaction, was assessed by ascending thin-layer chromatography (TLC) (silica gel Fluka, 706, 43–50 E.A.) by using the methanol/chloroform (9:1 *v*/*v*) and methylene chloride/chloroform (4:1 *v*/*v*) combination as the solvent system.

#### 3.1.1. Synthesis of 2a-b by the Action of Urea and Thiourea on Oxazine Derivative **1a**

A solution of **1a** (0.01 mole) and urea or thiourea (0.01 mole) in 30 mL of n-butanol was refluxed with 5 h. The solid that separated was crystallized from ethanol to give compounds **2a-b**.

#### 3.1.2. 2-(Tetrabromophthalimidomethyl-6-Phenyl-5-Cyano-1,3-Oxazino[4,5-e]-1,3-Pyrimidine-3-[H]-2,4-Dione (**2a**)

**2a**:yellow solid, m.p. 98–100 °C, yield 76%, IR (KBr) *ʋ* cm^−1^ 1720–1710 cm^−1^ (CO), 2230–2220 (CN), and 3380–3370 (NH). ^1^H NMR δ: 7.49–7.41 (m, 2H), 7.36–7.27 (m, 3H), 7.04 (d, *J* = 0.9 Hz, 1H), 5.35 (d, *J* = 6.0 Hz, 2H).^13^C NMR δ: 165.92, 162.30, 161.01, 159.31, 154.69, 134.59, 133.22, 129.09, 128.30 (d, *J* = 15.7 Hz), 126.96, 122.78, 117.42, 82.79, 50.51, 38.77. Elemental analysis calculated for C_22_H_9_N_5_O_5_Br_4_ (743); C:35.6; H:1.2; N:9.4. Found C:35; H:1; N: 9%.

#### 3.1.3. 2-(Tetrabromophthalimidomethyl-6-Phenyl-5-Cyano-1,3-Oxazino[4,5-e]-1,3-Pyrimidine-3-[H]-2-Thione-4-One (**2b**)

**2b**:brown solid, m.p. 150–152 °C, yield 79%, IR (KBr) *ʋ* cm^−1^ 1720–1710 (CO), 2230–2220 (CN), 1200–1190 (CS) and 3380–3370 (NH). ^1^H NMR δ: 7.49–7.41 (m, 2H), 7.36–7.27 (m, 3H), 7.04 (d, *J* = 0.9 Hz, 1H), 5.35 (d, *J* = 6.0 Hz, 2H).^13^C NMR δ: 182.32, 173.77, 165.92, 159.90, 158.70, 134.59, 133.15, 129.09, 128.37, 128.24, 126.91, 122.78, 118.70, 81.87, 50.49, 38.77. Elemental analysis calculated for C_22_H_9_N_5_O_4_SBr_4_ (759); C: 34.8; H: 1.2; N: 9.2. Found C: 34.2; H: 1.1; N: 8.9%.

#### 3.1.4. Synthesis of 2-(Tetrabromophalimidmethyl)-6-Phenyl-5-Yano-1,3-Oxazino[4,5-d]-1,2-Pyrazole2[H]-3-One (**3**)

A solution of 1a (0.01 mole) and hydrazine hydrate (0.01 mole) in 30 mL of n-butanol was refluxed for 5 h. The solid formed was collected and crystallized from benzene.

**3**:brown solid, m.p. 136–138 °C, yield 81%, IR (KBr) *ʋ* cm^−1^ 1715 cm^−1^ as the presence of CO, 2205 CN, and 3154–3350 NH, ^1^H NMR δ: 8.01 (s, 1H), 7.40–7.34 (m, 2H), 7.34–7.28 (m, 3H), 6.72 (d, *J* = 0.8 Hz, 1H), 4.72 (d, *J* = 6.0 Hz, 2H). ^13^C NMR δ: 167.13, 166.73, 159.97, 156.78, 142.21, 133.23, 131.92, 130.54, 129.00, 128.43, 128.38, 126.63, 124.96, 120.94, 120.87, 120.49, 80.82, 47.77, 37.81. Elemental analysis calculated for C_21_H_9_N_5_O_4_Br_4_ (715); C: 35.3; H: 1.3; N: 9.8. Found C: 34.4; H: 1.1; N: 9.5%.

#### 3.1.5. Synthesis of 4a-b by the Action of Urea and Thiourea on Oxazine Derivative **1b**

A solution of **1b** (0.01 mole) and urea or thiourea (0.01 mole) in 30 mL of n-butanol was refluxed for 5 h. The solid formed was crystallized from the proper solvent to give **4a-b**.

#### 3.1.6. Synthesis of 2-(Tetrabromophthalimidomethyl-6-Phenyl-5-Cyano-1,3-Oxazino[4,5-e]-1,3-Pyrimidine-3-[H]-2-One-5-Imide (**4a**)

**4a**:brown solid, m.p. 80–82 °C, crystalized from benzene, yield 72%, IR (KBr) *ʋ* cm^−1^ 2260 (CN), 3378 (NH) and 1780,1720 (CO). ^1^H NMR δ: 9.63 (s, 1H), 8.08 (s, 1H), 7.44–7.37 (m, 2H), 7.35–7.28 (m, 3H), 7.01 (d, *J* = 0.9 Hz, 1H), 5.35 (d, *J* = 6.0 Hz, 2H). ^13^C NMR δ: 166.53, 166.15, 163.32, 161.16, 154.22, 151.02, 140.50, 134.79 (d, *J* = 6.0 Hz), 134.13, 128.30 (d, *J* = 16.2 Hz), 126.88, 125.55, 123.97, 120.27, 119.71, 82.24, 40.21, 38.63. Elemental analysis calculated for C_22_H_10_N_6_O_4_Br_4_ (742); C: 35.6; H: 1.4; N: 11.4. Found C: 35.1; H: 1.2; N: 11%.

#### 3.1.7. 2-(Tetrabromophthalimidomethyl-6-Phenyl-5-Cyano-1,3-Oxazino[4,5-e]-1,3-Pyrimidine-3-[H]-2-Thione-4-Imide (**4b**)

**4b**:brown solid, m.p. 145–147 °C, crystalized from ethanol, yield 83%, IR (KBr) *ʋ* cm^−1^ 1278 cm^−1^ (CS), 2260 (CN), 3378 (NH), and 1780,1720 (CO). ^1^H NMR δ: 9.60 (s, 1H), 8.08 (s, 1H), 7.44–7.37 (m, 2H), 7.35–7.28 (m, 3H), 7.01 (d, *J* = 0.9 Hz, 1H), 5.35 (d, *J* = 6.0 Hz, 2H). ^13^C NMR δ: 180.40, 175.52, 166.53, 166.15, 160.35, 150.64, 140.50, 134.79 (d, *J* = 6.0 Hz), 134.12, 128.30 (d, *J* = 16.2 Hz), 126.88, 125.55, 123.34, 120.27, 119.71, 81.86, 48.24, 38.72. Elemental analysis calculated for C_22_H_10_N_6_O_4_Br_4_ (758); C: 34.9; H: 1.3; N: 11.1. Found C: 34.2; H: 1.2; N: 10. 9%.

#### 3.1.8. Synthesis of 2-(Tetrabromophalimidmethyl)-6-Phenyl-5-Yano-1,3-Oxazino[4,5-d]-1,2-Pyrazole2[H]-3-Imide (**5**)

A solution of 1b (0.01 mole) and hydrazine hydrate (0.01 mole) in 30 mL of n-butanol was refluxed for 5 h. Then, the crude was collected and crystallized from ethanol.

**5**:brown solid, m.p. 90–92 °C, yield 75%, IR (KBr) *ʋ* cm^−1^, 3350–3310 (NH), 2360 (CN), 1780, 1730 (CO), ^1^H NMR δ: 7.40–7.35 (m, 2H), 7.35–7.28 (m, 3H), 6.70 (d, *J* = 1.0 Hz, 1H), 5.02 (d, *J* = 7.0 Hz, 2H), 4.26 (t, *J* = 1.0 Hz, 2H), 3.01 (d, *J* = 2.0 Hz, 2H). ^13^C NMR δ: 172.60, 168.52, 159.98, 158.67, 131.92, 129.00, 128.59–128.30 (m), 128.08, 126.63, 120.49, 80.50, 47.78, 38.63, 35.63, 32.14. Elemental analysis calculated for C_21_H_10_N_6_O_3_Br_4_ (714); C: 35.3; H: 1.4; N: 11.8. Found C: 34.9; H: 1.2; N: 11.2%.

### 3.2. Biological Studies

#### 3.2.1. Antimicrobial Studies

All investigations were executed at the Biology department, Faculty of Science, Benha University, Egypt. The antimicrobial activities of all the synthesized molecules were determined in vitro, using the hole-plate and filter disc methodologies [33,34,35,36]. The investigated compounds were dissolved in 10% acetone (*v*/*v*). The width of the inhibition zone indicated the potency of the antimicrobial activity: (-) no antimicrobial activity, (+) mild activity with the diameter of the zones equal to (0.5–0.7 cm), (++) moderate activity with the diameter of the zones equal to (1.1–1.2 cm), and (+++) marked activity with the diameter of the zones equal to (1.6–1.8 cm). The results of the control samples are not included in Table 1, as they revealed a negative response.

#### 3.2.2. Anticancer

The cells were supplied by the Egyptian Holding Company for Biological Products and Vaccines (VACSERA) and then kept in the tissue culture unit. The growth of the cells was affected in RPMI-1640 medium, supplemented with 10% heat-inactivated FBS, 50 units/mL of penicillin, and 50 mg/mL of streptomycin, and maintained in a humidified atmosphere with 5% carbon dioxide [37,38]. The cells were maintained as monolayer cultures by serial sub-culturing, with cell culture reagents obtained from Lonza (Basel, Switzerland). The antitumor activities of the complexes were assessed against three cell lines MCF-7 for breast cancer, Ovcar-3 for ovarian cancer, and Hela for cervical cancer. In addition, all the results obtained were compared and validated to the LCC-MK2 normal cell line. In the literature, the sulforhodamine B (SRB) assay method was applied to determine the cytotoxicity, as described in [39]. Exponentially growing cells were collected using 0.25% Trypsin-EDTA and seeded in 96-well plates at 1000–2000 cells/well in RBMI-1640-supplemented medium. The cells were kept in the medium for 24 h and then incubated for 3 days with various concentrations of the copper complexes. Following 3 days of treatment, the cells were fixed with 10% trichloroethanoic acid for 1 h at 4 °C. Wells were stained for 10 min at room temperature with 0.4% SRBC dissolved in 1% acetic acid. The plates were air-dried for 24 h, and the dye was dissolved in Tris-HCl for 5 min with shaking at 1600 rpm. An ELISA microplate reader (ChroMate-4300, FL, USA) was used to assess each well’s optical density (O.D.) at 564 nm. The IC_50_ values were calculated from a Boltzmann sigmoidal concentration-response curve using the nonlinear regression fitting models (Graph Pad, Prism Version 9).

### 3.3. Computational Studies

#### 3.3.1. Molecular Docking Studies with MOE

Molecular operation environment software (MOE) was utilized to dock the complexes toward the MCF-7 breast (PDB = *3 KRR*), HeLa caspase3 (PDB = *V266*), and ovarian cancer targets (PDB = *3 W2S*). We used the docking protocol that was described in our previous work [13]. After the crystal structure was downloaded from the PDB www.rcsb.org (accessed on 12 July 2021), the water molecules, co-ligand, and metal ions were removed. The final form was obtained after 3D protonation and the correction process. The MOE site finder generated the active binding sites to create the dummy sites as the binding pocket. The default docking parameters were triangle matcher for replacing the molecule and London dG for rescoring the docking scores. The DFT-optimized structures of the compounds were used to generate the best five binding poses with flexible molecules rotation. The hydrogen bonds formed between the elastase and the investigated compound were used to rank the binding affinity and were presented as the free binding energy (S, kcal/mol). The higher negative values of the docking scores were presented along with 2D and 3D structures.

#### 3.3.2. Virtual Screening and Validation

Considering the three cell lines Ovcar-3, Hela, and MCF-7 used in the experimental procedures, the information regarding the protein targets was collected from the literature [22,23,40,41] and biological databases. The seven targets were selected for the virtual screening (Table 3). The 3-D coordinates of Caspase-3 (PDB ID-3KRR), Human Cyclin-Dependent Kinase 2 (CDK2, PDB ID-3QTR), Epidermal Growth Factor Receptor (EGFR, PDB ID-1XKK), Human B-Raf Kinase (PDB ID-3SKC), Human Estrogen Receptor Ligand-Binding Domain (PDB ID-1ERE), Human Serum Albumin (HSA, PDB ID-6WUW), and Human topoisomerase I (PDB ID-1EJ9) were collected from the database. The structures of the protein targets were optimized using the JAGUAR module [24] utilities present in MAESTRO (Schrödinger Release 2018-1: Maestro, Schrödinger, LLC, New York, NY, USA, 2018). Subsequently, the virtual screening between the target protein and studied inhibitors was performed using Autodock vina [42]. The best-docked conformations were statistically characterized by combining the free energy functional, the Lamarckian Genetic Algorithm, and the empirical force field [25]. The space of the grid dimension was set for 40 × 40 × 40 Å along with the XYZ directions with varied central coordinates, and a maximum efficiency range was used in the parameters for the optimum results.

#### 3.3.3. ADME and Pharmacophore Studies

The Lipinski’s rule of five (5) by Christopher A. Lipinski in 1997 is a thumb rule for evaluating drug-likeness and determining if an inhibitor with specific biological and pharmacological properties would be an orally active drug in the human body [21,25]. The rule states that a molecule can be orally absorbed/active if two (2) or more of these thresholds: molecular weight (Mw) of molecule < 500, octanol/water partition coefficient (ilog P) ≤ 5, number of hydrogen bond acceptors (nHBA) ≤ 10, number of hydrogen bond donors (nHBD) ≤ 5, and topological polar surface area (TPSA) < 40 Å^2^), are not violated.

#### 3.3.4. MD Simulations

After the analyses of the docking results, the complexes of **3a**, **4a**, and **5**, which were induced with HSA inhibitor, were selected for further studies using the GROMACS 2018-2 package [43], which was used to study their conformational dynamics under explicit water conditions for a 100 ns time scale. The OPLS-AA force field [44] was used to generate the topologies of the HSA protein in the docked complexes. The LigParGen server [45] generated the same force field potentials for the complex inhibitors. Afterward, the systems were immersed in the SPC/E water model [46,47] and neutralized by adding counter NA and CL ions. The further processing involved energy minimization using steepest descent and conjugate gradient algorithms, with a convergence criterion of 0.005 kcal/mol. The minimized systems were subjected to positions restraining and then equilibrated under NVT (constant volume) and NPT (constant pressure) ensemble conditions, each at a 100 ps time scale. The temperature of 300 K was maintained for the system using the Berendsen weak coupling method, and the pressure of 1 bar was maintained utilizing the Parrinello-Rahman barostat in the equilibration stage.

Furthermore, the final production stage was carried out using the LINCS algorithm. The generated trajectories were analyzed for the changes in the pattern of protein-inhibitor distances, H-bonds, RMSD, and Rg. Finally, the molecular mechanics Poisson-Boltzmann surface area (MM-PBSA) protocols implemented in the g_mmpbsa package [47] were used to calculate binding free energy between the HSA and inhibitors.

## 4. Conclusions

The present research strengthens the applicability of these compounds by encouraging anticancer and antibacterial drugs that could help medicinal chemists and pharmaceuticals further design and synthesize more effective drug candidates. Furthermore, with the collected and interpreted results, from oxazinethione, one can synthesize new pyrazoles and pyrimidine derivatives. According to ADME studies and molecular dynamics studies, compound **5** is the most potent for biological investigations, especially for Hela and ovarian cancer, with good pharmacokinetic properties showing high gastrointestinal absorption.

## Figures and Tables

**Table 1:** Biological screening activities of series of oxadizine-thione derivatives, activity (A) and minimum inhibitory concentration (MIC).

Compound Number	*A. flavus*		*E. coli*		*S. aureus*		*F. moniliform*	
	A	Mic	A	Mic	A	Mic	A	Mic
**2a**	-	-	-	-	+	125	+	125
**2b**	-	-	+	250	++	250	+	125
**3**	+	125	+	125	+	125	+	125
**4a**	+++	250	+	250	-	-	+	250
**4b**	-	-	+	250	++	250	+	125
**5**	+++	250	++	250	++	250	+	250

The zone width of inhibition indicates the potency of antimicrobial activity; (-) no activity, (+) mild activity with diameter zone of (0.5–0.7 cm), (++) moderate activity with diameter zone of (1.1–1.2 cm); (+++) marked with diameter zone of (1.6–1.8 cm).

**Table 2:** IC_50_ for tested compounds against different cell lines, in µg/mL.

Cpd.	MCF-7		Hela		Ovcar-3		LCC-MK2	
	IC_50_	±SD	IC_50_	+SD	IC_50_	±SD	IC_50_	±SD
**2b**	13.82	0.26	22.81	2.31	15.22	0.70	89.34	23.56
**3**	15.49	4.59	9.14	0.06	14.57	1.94	206.50	59.36
**4a**	21.70	3.09	12.88	0.10	15.86	0.50	68.91	2.37
**4b**	16.56	0.09	14.76	0.38	11.23	1.75	170.40	0.00
**5**	12.31	3.90	5.69	0.14	10.05	1.18	190.93	53.80

**Table 3:** Docking score and energies of all compounds with MCF-7 breast docking result of “*3 KRR*” protein.

Comp.	S	rmsd_refine	E_conf	E_place	E_score1	E_refine	E_score2
**2a**	−8.43	2.30	−212.78	−81.54	−12.39	−35.32	−8.43
	−8.17	1.29	−203.26	−82.90	−12.82	−40.22	−8.17
	−8.10	2.25	−194.54	−88.98	−11.17	−43.54	−8.10
	−8.09	1.36	−206.26	−72.74	−11.47	−33.41	−8.09
	−7.85	1.91	−207.36	−67.90	−11.13	−31.42	−7.85
**3**	−8.09	1.54	−65.96	−77.00	−11.74	−34.65	−8.09
	−8.28	1.97	−65.67	−53.76	−11.49	−28.68	−8.28
	−8.20	2.42	−72.23	−86.55	−10.34	−37.10	−8.20
	−8.15	1.66	−76.70	−119.31	−12.66	−48.38	−8.15
	−7.88	2.07	−76.97	−92.42	−11.00	−46.31	−7.88
**4a**	−8.06	2.27	−215.68	−87.53	−10.78	−39.39	−8.06
	−7.90	1.10	−212.32	−85.86	−10.69	−34.31	−7.90
	−7.89	1.12	−214.27	−94.83	−11.37	−33.22	−7.89
	−7.83	1.99	−210.89	−55.41	−10.83	−38.45	−7.83
	−7.80	1.63	−210.48	−78.77	−11.53	−35.98	−7.80
**4b**	−8.10	2.64	−192.81	−56.79	−9.91	−40.36	−8.10
	−7.75	1.64	−193.11	−82.44	−11.96	−34.88	−7.75
	−7.69	1.14	−191.25	−67.17	−11.13	−43.84	−7.69
	−7.67	2.93	−194.16	−84.16	−11.16	−44.49	−7.67
	−7.46	4.17	−177.93	−69.28	−10.53	−21.68	−7.46
**5**	−8.45	2.08	−76.73	−76.05	−11.80	−35.78	−8.45
	−8.27	1.65	−76.64	−60.46	−10.32	−30.34	−8.27
	−8.15	2.53	−86.74	−63.97	−10.31	−47.86	−8.15
	−8.09	2.16	−84.21	−75.90	−11.73	−45.81	−8.09
	−7.97	2.28	−84.96	−68.94	−10.87	−35.63	−7.97

**Table 4:** Docking score and energies of all compounds with HeLa caspase “*3 V266*” protein.

Comp.	S	rmsd_refine	E_conf	E_place	E_score1	E_refine	E_score2
**2a**	−6.22	1.41	−215.59	−55.68	−10.19	−37.97	−6.22
	−7.18	2.38	−215.00	−39.36	−10.47	−39.33	−7.18
	−6.71	2.48	−214.45	−85.06	−9.92	−39.51	−6.71
	−6.67	1.86	−214.32	−48.53	−10.00	−31.84	−6.67
	−6.49	1.20	−218.06	−81.95	−11.05	−28.54	−6.49
**3**	−7.45	1.46	−64.61	−63.24	−9.74	−37.05	−7.45
	−6.42	1.66	−71.66	−58.22	−10.24	−29.76	−6.42
	−6.40	1.50	−73.19	−82.55	−11.59	−36.54	−6.40
	−6.39	2.08	−74.87	−67.51	−10.52	−35.25	−6.39
	−6.38	1.98	−67.73	−73.49	−11.36	−28.50	−6.38
**4a**	−6.74	1.48	−224.62	−96.40	−10.43	−30.40	−6.74
	−6.59	4.54	−223.62	−41.68	−11.62	−34.24	−6.59
	−6.51	1.69	−222.06	−46.45	−10.35	−31.55	−6.51
	−6.45	1.28	−217.10	−42.55	−9.81	−27.20	−6.45
	−6.43	3.94	−223.58	−74.74	−10.32	−33.29	−6.43
**4a**	−7.15	2.11	−193.36	−72.65	−10.43	−38.75	−7.15
	−6.80	1.21	−201.45	−92.81	−10.69	−34.58	−6.80
	−6.61	4.02	−201.54	−68.46	−9.64	−36.48	−6.61
	−6.61	2.94	−199.69	−45.26	−9.68	−34.36	−6.61
	−6.44	1.98	−194.51	−61.61	−10.37	−31.49	−6.44
**5**	−7.91	2.58	−81.90	−45.97	−9.62	−43.31	−7.91
	−6.49	1.61	−83.08	−50.78	−9.51	−42.46	−6.49
	−6.39	1.52	−82.96	−67.06	−10.80	−34.79	−6.39
	−6.37	2.03	−85.56	−80.15	−9.61	−34.14	−6.37
	−6.26	2.93	−86.09	−61.42	−9.86	−31.70	−6.26

**Table 5:** Docking score and energies of all compounds with ovarian “*3 W2S*” protein.

Comp.	S	rmsd_refine	E_conf	E_place	E_score1	E_refine	E_score2
**2a**	−7.70	1.60	−219.01	−77.08	−10.27	−44.05	−7.70
	−7.19	2.26	−218.05	−78.25	−9.69	−39.51	−7.19
	−7.14	2.42	−215.81	−77.27	−10.93	−40.10	−7.14
	−7.00	1.80	−200.88	−62.91	−9.93	−25.82	−7.00
	−6.96	1.70	−224.02	−50.21	−9.71	−36.41	−6.96
**3**	−7.39	1.59	−70.23	−91.55	−9.50	−43.12	−7.39
	−7.54	2.85	−73.44	−84.32	−10.71	−45.10	−7.54
	−7.46	3.09	−78.20	−44.13	−10.22	−45.39	−7.46
	−7.21	2.26	−78.10	−57.97	−9.53	−45.34	−7.21
	−7.12	1.23	−70.06	−60.35	−9.94	−34.02	−7.12
**4a**	−7.66	1.10	−228.66	−99.53	−10.21	−44.05	−7.66
	−7.66	1.75	−214.85	−88.63	−10.97	−41.02	−7.66
	−7.06	1.13	−223.12	−92.78	−10.98	−34.17	−7.06
	−7.06	2.36	−205.71	−78.95	−9.73	−33.29	−7.06
	−6.74	1.14	−221.63	−46.92	−9.61	−32.15	−6.74
**4b**	−8.38	2.98	−201.86	−89.52	−10.94	−38.96	−8.38
	−7.36	1.18	−199.20	−93.88	−11.48	−39.58	−7.36
	−7.29	1.10	−205.78	−110.63	−11.67	−40.47	−7.29
	−7.07	2.36	−192.78	−64.95	−10.54	−39.50	−7.07
	−6.96	1.94	−196.57	−78.63	−10.34	−33.65	−6.96
**5**	−8.77	2.24	−86.58	−74.44	−12.15	−50.19	−8.77
	−7.69	1.78	−87.46	−83.46	−9.87	−39.94	−7.69
	−7.54	2.64	−73.58	−50.75	−10.56	−40.73	−7.54
	−7.46	1.71	−81.62	−70.61	−10.04	−40.33	−7.46
	−7.45	1.65	−88.79	−67.97	−10.21	−42.42	−7.45

**Table 6:** Docking interaction of all compounds with MCF-7 breast docking result of “*3 KRR*” protein.

Compound	Ligand	Receptor	Interaction	Distance E	(kcal/mol)
**2a**	O 13	CA GLY 858	H-acceptor	3.31	−1.6
**3**	N 44	NH1 ARG 980	H-acceptor	3.22	−1.1
**4a**	N 10	O LYS 857	H-donor	2.95	−1.2
	N 13	O LYS 857	H-donor	2.84	−0.5
	5-ring	CB LEU 855	pi-H	4.28	−0.7
	6-ring	CB LEU 855	pi-H	4.32	−0.7
**4b**	N 10	O LYS 857	H-donor	2.94	−0.3
	5-ring	CB LEU 855	pi-H	4.28	−0.4
	6-ring	CB LEU 855	pi-H	4.32	−0.4
**5**	N 43	NH1 ARG 980	H-acceptor	3.30	−1.9

**Table 7:** Docking interaction of all compounds with HeLa caspase “*3 V266*” protein.

Compound	Ligand	Receptor	Interaction	Distance E	(kcal/mol)
**2a**	Br 26	O GLY 122 (A)	H-donor	3.37	−2.5
	6-ring	NE ARG 64 (A)	pi-cation	4.79	−0.9
	6-ring	NE ARG 207 (A)	pi-cation	3.98	−0.9
	6-ring	NH1 ARG 207 (A)	pi-cation	3.28	−0.6
**3**	C 27	SG CYS 163 (A)	H-donor	3.85	−0.5
	6-ring	NE ARG 64 (A)	pi-cation	4.43	−1.9
**4a**	O 15	NE ARG 64 (A)	H-acceptor	3.36	−1.0
	O 15	NH2 ARG 64 (A)	H-acceptor	3.33	−2.4
	O 15	NE ARG 207 (A)	H-acceptor	3.10	−1.6
**4b**	Br 28	O GLY 122 (A)	H-donor	3.44	−0.5
	6-ring	SG CYS 163 (A)	pi-H	4.18	−0.7
	6-ring	NE ARG 207 (A)	pi-cation	3.71	−1.1
**5**	Br 15	O GLY 60 (A)	H-donor	3.50	−0.8
	O 30	CA TRP 206 (A)	H-acceptor	3.42	−0.5
	O 30	N ARG 207 (A)	H-acceptor	3.14	−2.4
	6-ring	NE ARG 64 (A)	pi-cation	4.89	−0.9

**Table 8:** Docking interaction of all compounds with ovarian “*3 W2S*” protein.

Compound	Ligand	Receptor	Interaction	Distance E	(kcal/mol)
**2a**	C 30	O ARG 841 (A)	H-donor	3.24	−0.5
	6-ring	CD ARG 841 (A)	pi-H	3.75	−1.0
**3**	No measurable interactions				
**4a**	6-ring	CD ARG 841 (A)	pi-H	3.92	−0.7
**4b**	C 31	O ARG 841 (A)	H-donor	3.38	−0.8
	6-ring	CG1 VAL 726 (A)	pi-H	4.50	−0.5
	6-ring	CD ARG 841 (A)	pi-H	3.74	−0.7
**5**	N 24	O GLY 724 (A)	H-donor	2.85	−2.8
	N 43	NZ LYS 745 (A)	H-acceptor	3.00	−10.2
	6-ring	CA GLY 721 (A)	pi-H	4.24	−0.9
	6-ring	N ALA 722 (A)	pi-H	4.79	−0.7

**Table 9:** Physicochemical Properties.

Comp.	2b	3	4a	4b	5
Formula	C_22_H_9_Br_4_N_5_O_4_S	C_21_H_10_Br_4_N_6_O_3_	C_22_H_11_Br_3_N_6_O_4_	C_22_H_11_Br_3_N_6_O_3_S	C_21_H_9_Br_4_N_5_O_4_
Molecular weight	759.02 g/mol	713.96 g/mol	663.07 g/mol	679.14 g/mol	714.94 g/mol
Num. heavy atoms	36	34	35	35	34
Num. arom. heavy atoms	12	12	12	12	12
Fraction Csp3	0.14	0.14	0.14	0.14	0.14
Num. rotatable bonds	3	3	3	3	3
Num. H-bond acceptors	7	7	8	7	7
Num. H-bond donors	1	2	2	2	1
Molar Refractivity	160.73	151.53	149.22	156.42	148.13
TPSA	156.31 Å^2^	131.00 Å^2^	148.07 Å^2^	163.09 Å^2^	124.22 Å^2^
**Lipophilicity**					
Log *P*_o/w_ (iLOGP)	2.81	2.19	2.09	2.50	2.40
Log *P*_o/w_ (XLOGP3)	4.53	4.14	3.33	3.93	4.05
Log *P*_o/w_ (WLOGP)	2.98	3.06	2.50	2.67	2.61
Log *P*_o/w_ (MLOGP)	3.88	4.56	3.76	3.72	4.16
Log *P*_o/w_ (SILICOS-IT)	6.82	5.57	4.81	6.27	5.44
Consensus Log *P*_o/w_	4.20	3.91	3.30	3.82	3.73
**Water Solubility**					
Log *S* (ESOL)	−7.45	−6.94	−6.10	−6.58	–6.89
Solubility	2.70 × 10^−5^ mg/mL; 3.56 × 10^−8^ mol/L	8.24 × 10^−5^ mg/mL; 1.15 × 10^−7^ mol/L	5.21 × 10^−5^ mg/mL; 7.86 × 10^−7^ mol/L	1.78 × 10^−5^ mg/mL; 2.62 × 10^−7^ mol/L	9.27 × 10^−5^ mg/mL; 1.30 × 10^−7^ mol/L
Class	Poorly soluble	Poorly soluble	Poorly soluble	Poorly soluble	Poorly soluble
Log *S* (Ali)	−7.53	−6.60	−6.12	−7.05	−6.36
Solubility	2.22 × 10^−5^ mg/mL; 2.92 × 10^−8^ mol/L	1.80 × 10^−4^ mg/mL; 2.52 × 10^−7^ mol/L	5.07 × 10^−4^ mg/mL; 7.65 × 10^−7^ mol/L	5.99 × 10^−5^ mg/mL; 8.83 × 10^−8^ mol/L	3.10 × 10^−4^ mg/mL; 4.34 × 10^−7^ mol/L
Class	Poorly soluble	Poorly soluble	Poorly soluble	Poorly soluble	Poorly soluble
Log *S* (SILICOS-IT)	−9.20	−9.04	−8.44	−8.62	−8.90
Solubility	4.76 × 10^−7^ mg/mL; 6.27 × 10^−10^ mol/L	6.44 × 10^−7^ mg/mL; 9.02 × 10^−10^ mol/L	2.43 × 10^−6^ mg/mL; 3.66 × 10^−9^ mol/L	1.64 × 10^−6^ mg/mL; 2.42 × 10^−9^ mol/L	9.02 × 10^−7^ mg/mL; 1.26 × 10^−9^ mol/L
Class	Poorly soluble	Poorly soluble	Poorly soluble	Poorly soluble	Poorly soluble
**Pharmacokinetics**					
G.I. absorption	Low	High	Low	Low	High
BBB permeant	No	No	No	No	No
P-gp substrate	No	No	No	No	No
CYP1A2 inhibitor	Yes	Yes	No	Yes	Yes
CYP2C19 inhibitor	No	No	No	No	No
CYP2C9 inhibitor	No	No	No	No	No
CYP2D6 inhibitor	No	No	No	No	No
CYP3A4 inhibitor	No	No	No	No	No
Log *K*_p_ (skin permeation)	−7.71 cm/s	−7.72 cm/s	−7.98 cm/s	−7.65 cm/s	−7.79 cm/s
**Drug-likeness**					
Lipinski	Yes; 1 violation: MW > 500	No; 2 violations: MW > 500, MLOGP > 4.15	Yes; 1 violation: MW > 500	Yes; 1 violation: MW > 500	No; 2 violations: MW > 500, MLOGP > 4.15
Ghose	No; 2 violations: MW > 480, MR > 130	No; 2 violations: MW > 480, MR > 130	No; 2 violations: MW > 480, MR > 130	No; 2 violations: MW > 480, MR > 130	No; 2 violations: MW > 480, MR > 130
Veber	No; 1 violation: TPSA > 140	Yes	No; 1 violation: TPSA > 140	No; 1 violation: TPSA > 140	Yes
Egan	No; 1 violation: TPSA > 131.6	Yes	No; 1 violation: TPSA > 131.6	No; 1 violation: TPSA > 131.6	Yes
Muegge	No; 2 violations: MW > 600, TPSA > 150	No; 1 violation: MW > 600	No; 1 violation: MW > 600	No; 2 violations: MW > 600, TPSA > 150	No; 1 violation: MW > 600
Bioavailability Score	0.55	0.17	0.55	0.55	0.17
**Medicinal Chemistry**					
PAINS	0 alert	0 alert	0 alert	0 alert	0 alert
Brenk	4 alerts: halogenated_ring_1, halogenated_ring_2, phthalimide, thiocarbonyl_group	4 alerts: halogenated_ring_1, halogenated_ring_2, imine_1, phthalimide	3 alerts: halogenated_ring_1, imine_1, phthalimide	4 alerts: halogenated_ring_1, imine_1, phthalimide, thiocarbonyl_group	3 alerts: halogenated_ring_1, halogenated_ring_2, phthalimide
Lead-likeness	No; 2 violations: MW > 350, XLOGP3 > 3.5	No; 2 violations: MW > 350, XLOGP3 > 3.5	No; 1 violation: MW > 350	No; 2 violations: MW > 350, XLOGP3 > 3.5	No; 2 violations: MW > 350, XLOGP3 > 3.5
Synthetic accessibility	4.61	4.58	4.74	4.66	4.52

**Table 10:** Pharmacokinetic properties.

Model Name	Pharmacokinetic Properties				
Water solubility (log mol/L)	−5.621	−4.823	−4.608	−4.749	−5.483
Caco2 permeability (log Papp in 10^−6^cm/s)	0.69	0.621	0.001	0.58	0.665
Intestinal absorption (human) (% Absorbed)	83.586	84.559	80.732	82.507	84.596
Skin Permeability (log Kp)	−2.826	−2.8	−2.779	−2.782	−2.863
P-glycoprotein substrate	Yes	Yes	Yes	Yes	Yes
P-glycoprotein I inhibitor	Yes	Yes	Yes	Yes	Yes
P-glycoprotein II inhibitor	Yes	Yes	No	No	Yes

**Table 11:** Oral toxicity prediction results for all selected compounds.

Compound	2b	3	4a	4b	5
Predicted LD_50_	10,000 mg/kg	1000 mg/kg	1000 mg/kg	1168 mg/kg	300 mg/kg
Predicted ToxicityClass *	6	4	4	4	3
Average similarity	34.04%	31.94%	35.06%	33.99%	32.14%
Prediction accuracy:	23%	23%	23%	23%	23%
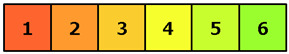 *					

**Table 12:** List of virtual screening-based parameters for the selected drug targets against the studied anticancer inhibitors (green color selected for MD simulations).

S. No	Inhibitors	Free Energy of Binding (kcal/mol)						
		Caspase-3	Human Cyclin-Dependent Kinase 2 (CDK2)	Epidermal Growth Factor Receptor (EGFR)	Human B-Raf Kinase	Human Estrogen Receptor Ligand-Binding Domain	Human Serum Albumin	Human Topoisomerase I
1	**2a**	−6.7	−8.7	−9.1	−8.5	−8.1	−8.6	−8.1
2	**2b**	−6.7	−7.5	−8.2	−8.2	−8.2	−8.2	−7.9
3	**3**	−6.4	−8.0	−7.9	−9.2	−7.6	−9.8	−8.2
4	**4a**	−6.6	−9.0	−8.1	−8.3	−8.5	−9.9	−8.1
5	**4b**	−6.9	−7.4	−8.5	−8.5	−7.8	−8.4	−8.2
6	**5**	−6.7	−8.8	−8.9	−9.2	−7.7	−9.3	−8.2

**Table 13:** List of MM-PBSA-based generated energy parameters for the studied anticancer inhibitors complexed HSA.

S. No	Docked Complex	MM-PBSA-Based Calculated Energies (kJ/mol)			
		Van Der Waals	Electrostatic	SASA	Binding e Energy
1	**3**	−308.232	−22.682	−22.504	−375.922
2	**4a**	−244.065	−29.981	−23.239	−320.525
3	**5**	−259.583	−41.077	−23.113	−346.887

## Data Availability

Not available.

## Sample Availability

Not available.

## Abbreviations

MIC	minimum inhibitory concentration
MOE	Molecular Operating Environment
MD	Molecular Dynamics
*A. flavus*	*Aspergillus flavus*
*E. coli*	*Escherichia coli*
*S. aureus*	*Staphylococcus aureus*
*F. moniliform*	*Fusarium moniliform*
PDB	Protein Data Bank
ADME	absorption, distribution, metabolism, and excretion
